# The Effect of Robot Attentional Behaviors on User Perceptions and Behaviors in a Simulated Health Care Interaction: Randomized Controlled Trial

**DOI:** 10.2196/13667

**Published:** 2019-10-04

**Authors:** Deborah L Johanson, Ho Seok Ahn, Bruce A MacDonald, Byeong Kyu Ahn, JongYoon Lim, Euijun Hwang, Craig J Sutherland, Elizabeth Broadbent

**Affiliations:** 1 Department of Psychological Medicine Faculty of Medical and Health Sciences The University of Auckland Auckland New Zealand; 2 Centre for Automation and Robotic Engineering Science The University of Auckland Auckland New Zealand; 3 Department of Electrical, Computer, and Software Engineering Faculty of Engineering The University of Auckland Auckland New Zealand

**Keywords:** robotics, health care robotics, social interaction, engagement, social intelligence

## Abstract

**Background:**

For robots to be effectively used in health applications, they need to display appropriate social behaviors. A fundamental requirement in all social interactions is the ability to engage, maintain, and demonstrate attention. Attentional behaviors include leaning forward, self-disclosure, and changes in voice pitch.

**Objective:**

This study aimed to examine the effect of robot attentional behaviors on user perceptions and behaviors in a simulated health care interaction.

**Methods:**

A parallel randomized controlled trial with a 1:1:1:1 allocation ratio was conducted. We randomized participants to 1 of 4 experimental conditions before engaging in a scripted face-to-face interaction with a fully automated medical receptionist robot. Experimental conditions included a self-disclosure condition, voice pitch change condition, forward lean condition, and neutral condition. Participants completed paper-based postinteraction measures relating to engagement, perceived robot attention, and perceived robot empathy. We video recorded interactions and coded for participant attentional behaviors.

**Results:**

A total of 181 participants were recruited from the University of Auckland. Participants who interacted with the robot in the forward lean and self-disclosure conditions found the robot to be significantly more stimulating than those who interacted with the robot in the voice pitch or neutral conditions (*P*=.03). Participants in the forward lean, self-disclosure, and neutral conditions found the robot to be significantly more interesting than those in the voice pitch condition (*P*<.001). Participants in the forward lean and self-disclosure conditions spent significantly more time looking at the robot than participants in the neutral condition (*P*<.001). Significantly, more participants in the self-disclosure condition laughed during the interaction (*P*=.01), whereas significantly more participants in the forward lean condition leant toward the robot during the interaction (*P*<.001).

**Conclusions:**

The use of self-disclosure and forward lean by a health care robot can increase human engagement and attentional behaviors. Voice pitch changes did not increase attention or engagement. The small effects with regard to participant perceptions are potentially because of the limitations in self-report measures or a lack of comparison for most participants who had never interacted with a robot before. Further research could explore the use of self-disclosure and forward lean using a within-subjects design and in real health care settings.

## Introduction

### Background

The use of social robots in home and health care environments is fast becoming a reality [1,2]. Although much robotics research is focused on the technical capabilities of robots, it is also important that research considers the behaviors of robots to ensure that interactions between humans and robots are successful. Consideration of robot social behaviors is perhaps even more salient when considering health care robots, which may be interacting with potentially vulnerable individuals on a daily basis. For interactions between patients and health care robots to be successful, these robots will need to behave in a way that is not only useful but also acceptable and comfortable [3]. One way to inform research investigating appropriate robot social behaviors in human-robot interactions is to consider the social behaviors that lead to successful human interactions.

Attentional behaviors are an important group of human social behaviors, fundamental to ensuring successful interactions. Attentional behaviors include the ability to not only demonstrate attention but also to engage and maintain the attention of others. As put by Zhao et al [4], “mutual attentiveness leads to an experience of connectedness” (p. 515). Given the importance of attention in human social interactions, it is critical that researchers investigating the social aspects of health care robots explore human attentional research to inform potential research within this area. A number of researchers have, in fact, taken this approach, researching several key human attentional behaviors within the context of human-robot interactions. One of these key behaviors is eye gaze.

Eye gaze is crucial to establish human joint attention, which, in turn, is a critical aspect of human learning, communication, and social interaction [5,6]. In a health care context, the appropriate use of eye gaze by a physician has been found to be associated with increased patient satisfaction and increased patient ratings of physician empathy, physician attention, and physician warmth [7,8]. In human-robot interactions, robot eye gaze has been found to increase human attention and engagement and facilitate comprehension of robot communication [9-11].

Despite growing research into the importance of robot attentional behaviors, a number of key human attentional behaviors are yet to be explored within the context of human-robot interaction, especially in health care. Three such attentional behaviors include the use of self-disclosure, voice pitch changes, and a forward lean. These attentional behaviors have been found to be important in human social interactions and in interactions between patients and health care professionals. The following section of this paper describes previous research examining the use of self-disclosure, voice pitch, and forward lean in human interactions. In the instance that research has been done exploring one of these behaviors in the context of human-robot interactions, this is presented.

#### Self-Disclosure

Self-disclosure refers to the *act of revealing personal information about oneself to another* and is recognized as central to the process of building close relationships [12]. Research into the use of self-disclosure in human interactions has found that self-disclosure is more effective when negatively skewed. For example, Zhao et al [4] found that in conversations between 2 individuals, previously unknown to each other, self-disclosure was often in the form of personally negative statements (eg, “I’m always late for the bus”), and that these statements were then often met with similarly negative statements (“Me too!”). The authors state that this seemingly superficial conversation tool increased mutual gaze among participants and often lead to more intimate conversation. Failure to reciprocate negative self-disclosure can lead to decreases in feelings of rapport [13].

The study of robot self-disclosure and its effect on human-robot interactions is limited. In the research that has been undertaken so far, the use of self-disclosure by a robot has been shown to increase users’ ratings of a robot’s agency and experience [12], stabilize users’ anxiety about a robot’s communication capacity [14], increase users’ perceptions of a robot’s likability, and decrease users’ feelings of control [15]. No research to date has examined the effect of robot self-disclosure on human engagement or attention. Patient attention and engagement are critical to patient satisfaction and adherence; therefore, it is important that further research is conducted to investigate whether self-disclosure from a robot can influence attention to the robot in a health care application [16].

#### Forward Body Lean

It is equally important to examine nonverbal attentional behaviors as it is to examine verbal attentional behaviors. Eye gaze is one example of an important nonverbal attentional behavior, used by a listener to engage and demonstrate attention toward a speaker.

The use of a forward body lean by a listener is another salient way to display attention, interest, and agreeance toward a speaker [17-19]. Leaning forward toward another individual to display attention is an almost automatic behavior and is even found to be used in those communicating through sign language [19].

Given the importance of using a forward body lean to demonstrate attention in human interactions, it is perhaps surprising to note that there has been no research undertaken examining the effect of robot forward body lean, in the context of human-robot interactions, on any outcome variables. There has been some research on robot forward neck tilt, which*,* when used alongside expressive facial movements, has been shown to aid human recognition of robot emotions [20], human comprehension of robot behaviors [21], and facilitate turn-taking [21]. The robot used in these studies, however, was not a health care robot and was made up of a *head* and *neck* with no *body*. The lack of research on forward body lean by any robot, particularly a health care robot, represents an important gap in our knowledge.

#### Voice Pitch Changes

A person’s voice pitch, or in other words, how low or high a voice is in frequency [22], conveys a range of information to others, such as gender and emotional affect [22,23]. Voice pitch has even been found to influence perceptions of attractiveness, with research finding that men rate woman with high-pitched voices as more attractive than those with low-pitched voices [24]. Research in the area of verbal communication has found that individuals can determine the personality traits of others with considerable accuracy, purely through patterns of speech, such as speed and voice pitch [25].

Voice pitch and voice pitch changes are an important part of attending behaviors and essential for communication [26]. Voice pitch changes allow a speaker to place emphasis on certain words, infuse emotion into specific phrases, and influence comprehension through the use of inflection (eg*,* in the case of a statement or question) to initiate and sustain the attention of others [26,27]. Therefore, key is the use of voice pitch changes in attention and communication, for example, individuals often exaggerate voice pitch changes when storytelling to hold the attention of their audience [28]. Other research investigating voice pitch in human interactions provides further support for the effect of voice pitch on attention. A recent study found that retention of content in long-term memory was higher when individuals listened to voices using high and low voice pitches, as opposed to a medium voice pitch [29]. This finding was independent of whether individuals listened to natural voices or voices that had been manipulated. These studies provide a rationale for examining the effects of both high and low robot voice pitch changes on human-robot interactions.

Although previous research in human-robot interactions has investigated robot voice–related variables, such as robot voice gender [30], robot voice age [31], and robot voice human likeness [32], in regard to user outcomes, only 1 study to date has explored the effect of robot voice pitch in the context of human-robot interactions. This study by Niculescu et al [22] compared a robot with a high voice pitch against a robot with a low voice pitch, finding that the robot with the high voice pitch was rated by participants as significantly more likable and attractive, with a better *personality*. In addition, the interaction with the robot with the high-pitched voice was rated more exciting, entertaining, and enjoyable.

### Justification for Research in Health Care

A model of robot-patient interaction proposes that behaviours that are important in physician-patient communication may also be important in communication between healthcare robots and patients [33]. Self-disclosure, forward lean, and voice pitch are key behaviors in good physician-patient communication, as detailed below. Therefore, these behaviors are likely to be important in interactions between health care robots and patients to establish a good rapport. However, research is needed to test this hypothesis.

#### Physician-Patient Communication Theory

Doctor-patient or physician-patient communication theory posits that the way in which a physician communicates with a patient is just as important as the information they provide for patient outcomes. Physician-patient communication is key to building rapport with patients and central to the delivery of appropriate health care [33,34]. Effective physician verbal and nonverbal communication has been found to decrease patient anxiety and psychological distress [35,36], facilitate patient understanding of medical information [34], and increase patient satisfaction [33].

The central goals of effective physician-patient communication are to facilitate the establishment of rapport, enable exchange of health-related information, and promote patient involvement in treatments plans and health-related decision making [33,34,36,37]. To accomplish these goals, a physician needs to act in a way that demonstrates their attention to the patient. In fact, the Toronto Consensus Agreement recommends that all physicians should actively attend to patients to encourage full expression of health concerns, without interruption or premature closure of conversation [37].

#### Forward Lean in Health Care

As previously discussed, use of a forward body lean is 1 way to demonstrate attention and establish rapport. In a health care context, forward lean is used by clinicians to demonstrate attention or *active listening* to patients [29]. Indeed, a study by Sharpley and Sagris [38] found that the use of forward lean by a counsellor was associated with increased client ratings of rapport. In research examining physician-patient interactions, the use of forward lean by a physician was found to be associated with positive patient perceptions of physician empathy, respect, and genuineness [39]. A systematic review by Beck et al [40] found physician forward lean to be among the behaviors that were significantly associated with increases in physician-patient rapport, increased patient satisfaction, and increased patient understanding. Owing to the fact that forward lean is used as a way of demonstrating attention across many cultures, its use is recommended to health care professionals as appropriate for use with most patients [29].

#### Self-Disclosure in Health Care

Although many physicians are trained not to self-disclose, a recent systematic review found self-disclosure was routinely used by physicians in clinical practice [41]. This same review found that, when used appropriately, self-disclosure by a physician had the potential to significantly increase patient satisfaction and physician-patient rapport. In clinicians, appropriate use of self-disclosure usually involves the disclosure of personal information to a patient that is relevant to the therapeutic process [42]. In this way, self-disclosure is able to demonstrate to a patient that they have been heard, as well as creating a feeling of shared experience.

#### Voice Pitch in Health Care

In clinicians, voice pitch (or voice tone) is used as a way to demonstrate attention and empathy to patients [29]. In a review of doctor-patient communication [43], researchers found that patients were less satisfied with their consultation when their physician used a negative voice tone or had tension in their tone. In a later study of surgeon’s malpractice history, surgeons who were perceived to have a dominant voice tone (ie, deep and loud) were more likely to have been sued by patients compared with surgeons with a less dominant voice pitch (ie, higher and softer) [44].

### This Study

The human medical receptionist is the first point of contact for patients seeking medical assistance in specialist and general practice clinics [45]. As such, the communication behaviors of receptionists can significantly impact patients [46,47]. It is therefore important that researchers investigate which aspects of human behaviors are appropriate for medical receptionist robots to ensure interactions are successful.

As detailed above, a number of human attentional behaviors have yet to be fully explored within the context of health care human-robot interactions. In regard to self-disclosure, a handful of studies have investigated the effect of robot self-disclosure on user outcomes such as anxiety [15], perceived robot likability [14], and perceived robot mind attribution [12]. However, no studies have investigated the effect of robot self-disclosure on any user attentional outcomes, such as engagement (attention), perceived robot attention, and perceived robot empathy, or user attentional behaviors in health care. In regard to forward body lean, no study could be found investigating the effect of robot forward body lean on user attentional behaviors or any other outcome.

Although 1 study has compared the effect of a robot using a high voice pitch against the effect of a robot using a low voice pitch, no study could be found investigating the effect of a robot using both high and low voice pitch changes within a single human-robot interaction. Given the importance of self-disclosure, forward lean, and voice pitch changes in both human social interactions and interactions between health care professionals and patients, it is critical that robotics researchers investigate these attentional behaviors in interactions between robots and human users. Research in this area could potentially inform the future design and implementation of not only social robots but also, more specifically, health care robots.

### Study Objectives and Hypotheses

The aim of this research was to investigate the effects of robot self-disclosure, forward lean, and voice pitch changes on user perceptions and attentional behaviors in a health care context. We hypothesized that, compared with a neutral condition, these robot behaviors would increase participants’ perceptions of engagement, robot empathy, and robot attention and increase participants’ own attentional behaviors.

## Methods

### Experimental Design

**Figure 1:**
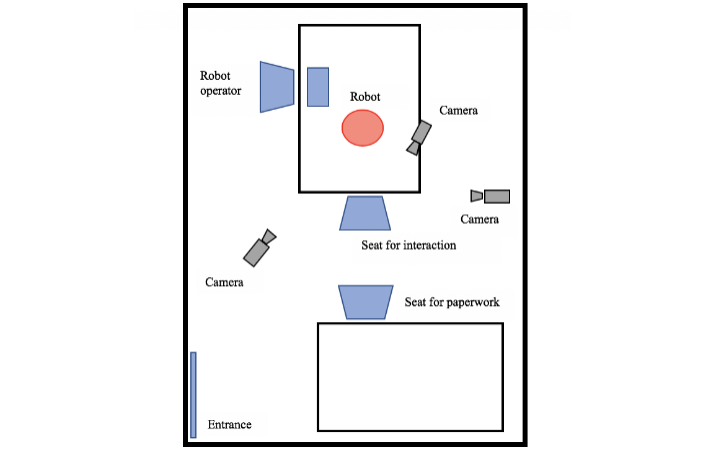
Experimental setup.

A between-subjects experimental study was conducted at the University of Auckland, Auckland, New Zealand. Participants completed baseline measures before being randomized to 1 of the 4 experimental groups (ie, self-disclosure, voice pitch, forward lean, or neutral condition). Following the interaction with the robot, postinteraction measures were completed. The interaction between each participant and the robot was video recorded from 3 different angles to allow for coding of participant behaviors (Figure 1).

### Participants

Participants were recruited through flyers posted at the University of Auckland’s campuses and through emails to students. Eligibility criteria were being 16 years of age or older and fluent in English. Written informed consent was obtained.

### The Robot

**Figure 2:**
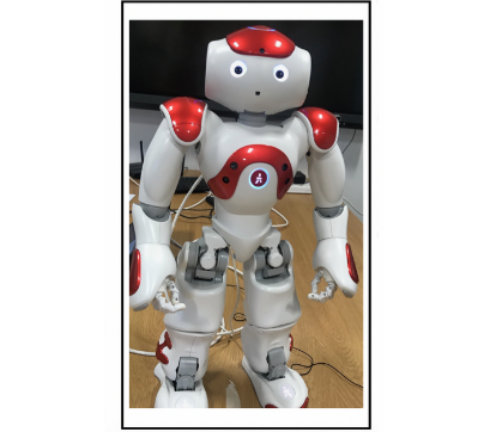
Nao: the medical receptionist robot.

A Nao robot (Softbank, Japan) was chosen for this study, as it was able to meet the requirements of the experimental conditions in regard to forward body lean, spoken conversation, and voice pitch changes. The Nao robot is an autonomous, programmable, humanoid robot, able to perform a variety of physical movements and speech patterns (Figure 2). A single Nao robot was used, with each participant interacting with the robot on an individual basis.

### Procedures

Once randomized to 1 of the 4 experimental conditions, participants were asked to imagine that they were attending their current general practitioner’s office, and the Nao robot was the robot receptionist. The participant was provided with a script for use during the interaction with the robot. This script was identical for all conditions and instructed participants to undertake a variety of tasks during their interaction with the Nao robot (Multimedia Appendix 1). The tasks undertaken during the interaction included activities that account for over 90% of the interactions between human medical receptionists and patients, such as checking in for a doctor’s appointment and collecting a prescription [47]. Once the robot operator selected the appropriate experimental interaction, the robot introduced itself as “Nao the Receptionist Robot” before enquiring as to how it could be of help to the participant. The Nao robot used identical speech responses and prompts across all conditions, with the exception of the self-disclosure condition.

**Figure 3:**
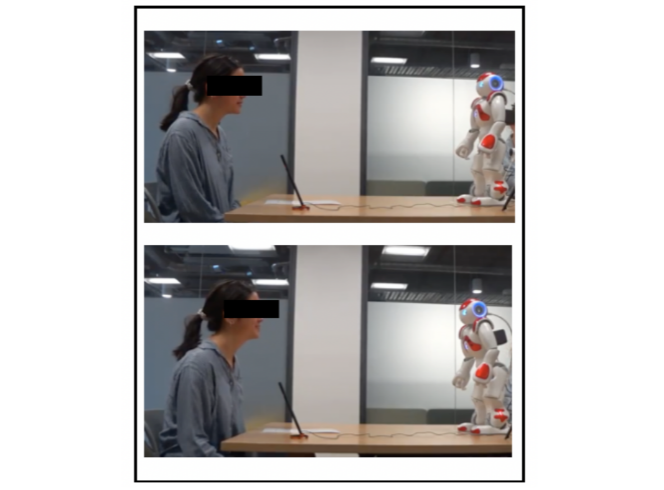
The Nao robot—from a neutral standing to forward lean position.

In the self-disclosure condition, the Nao robot stated, “I’m a little nervous about this task” after introducing itself to the participant. In addition, when a participant advised that they could not remember the name of the doctor they were seeing (as per the Participant Scenario Information Sheet, Multimedia Appendix 1), the robot stated, “no problem, I forget things too sometimes” before continuing the scripted interaction. In the forward body lean condition, the Nao robot leaned (approximately 20°) forward toward a participant when he or she was speaking, maintaining a neutral standing position during the rest of the interaction (Figure 3).

In the voice pitch condition, the Nao robot both *increased and decreased* its voice pitch within the single interaction. The Nao robot *decreased* its voice pitch (by 15%) when apologizing and advising a participant that their script was not available for collection. This lowering of robot voice pitch was intended to display robot *sadness* to the participant in relation to being unable to assist with their request. Studies investigating vocal emotion recognition have shown that sadness is best recognized through voice alone when a lower voice pitch is used [48,49] (compared with a neutral). The robot *increased* its voice pitch (by 15%) when advising a participant, it was “no problem” in regard to helping the participant with the name of the doctor they were seeing and when stating “I hope you have a nice day” at the end of the interaction. This increase in robot voice pitch was intended to display robot *happiness* to the participant in relation to being able to assist the participant and in wishing them a good day. Studies investigating vocal emotion recognition have shown that happiness is best recognized through voice alone when a higher voice pitch is used [48,49] (compared with a neutral). Finally, in the neutral condition, the Nao robot maintained a neutral standing position and neutral voice pitch throughout the interaction, with no self-disclosure.

### Measures

#### Participant Engagement

A Likert scale was developed using an adaption of the *stimulation* items from the McGill Friendship Questionnaire [50], along with an adaption of the engagement items used in the human-robot engagement study by Snider et al [51] (Multimedia Appendix 2). The Cronbach alpha for the combination of these Likert items was found to be .86, showing excellent reliability. Therefore, the scores from these items were added to create a total *participant engagement* score.

In addition, pair-choice items were developed using an adaption of the *stimulation* (paired) items from the AttrakDiff user experience tool created by Hassenzahl et al [52]. All pair-choice items were analyzed separately.

Adaptions of both the McGill Friendship Questionnaire and the AttrakDiff user experience tool have been used previously in human-robot interaction research [22,53].

#### Perceived Robot Attention

No scale was found measuring human perceptions of robot attention or attentiveness. Therefore, a new measure was created using an adaption of the *stimulation* items from the McGill Friendship Questionnaire [50], along with an adaption of the engagement items used in the human-robot interaction study by Snider et al [51] (Multimedia Appendix 2). The Cronbach alpha for this measure was .89, showing excellent reliability. Thus, the scores from all items were added to create a total *perceived robot attention* score.

#### Perceived Robot Empathy

An adaption of the McGill Friendship Questionnaire [50] was used along with an adaption of the consultation and relational empathy measure [54] (Multimedia Appendix 2). The consultation and relational empathy measure assesses patient perceived empathy in relation to clinical encounters and has been found to be both valid and reliable across clinical settings [55,56]. An adaption of the McGill Friendship Questionnaire was used in research investigating perceived robot empathy by Leite et al [53]. The Cronbach alpha for this combined measure was found to be .82. After removal of the item, “I think Nao had fun during this interaction,” Cronbach alpha increased to .89. Therefore, the scores from all remaining items were added to create a total *perceived robot empathy* score.

#### Observer Ratings

The video ratings were used in addition to self-reports to measure participant attention and engagement. Eye gaze and forward lean were used to measure attention, and smiling and laughter were used to measure engagement.

**Figure 4:**
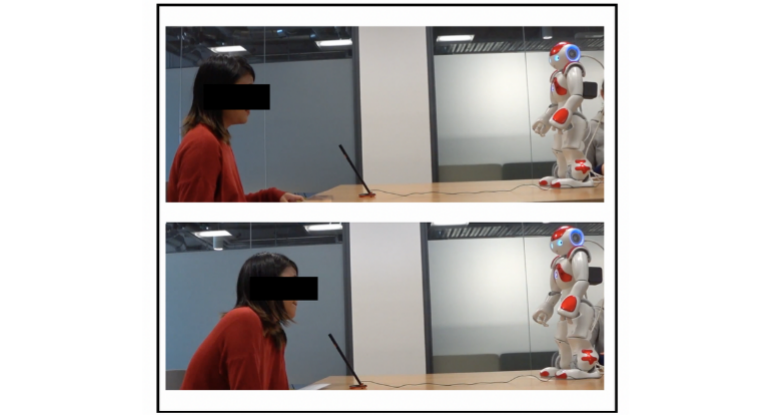
Participant moving from neutral to forward lean position.

Video recordings were viewed to determine the overall time (in seconds) that each participant spent looking at the Nao robot during the interaction. Using this time and the total interaction time, a percentage was able to be determined in regard to the time spent looking at the Nao robot. Each video recording was coded in regard to whether or not a participant leant toward the Nao robot during the course of the interaction. A *forward lean* was identified by placing a visual marker on the participants’ midback (during the video playback) when they had settled into the experimental chair and then watching to see if the participant leaned toward the robot, forward from the marker, at any time during the interaction (Figure 4). As many participants sat down and immediately leant forward (resting their forearms on the table), this posture was not identified as a forward lean unless active forward leaning past this *neutral* point was identified. In addition, many participants leant forward into the microphone (that was positioned on the desk) when they spoke, and therefore, this forward leaning movement was disregarded.

Each of the video recordings was then viewed to determine the overall time (in seconds) that each participant spent smiling during the interaction with the Nao robot. As with time spent looking at the robot, this time was used to give a percentage of time spent smiling at the robot for each participant. Each video recording was also coded in regard to whether or not a participant laughed during the course of the interaction with the Nao robot. The coding in regard to whether or not a participant *laughed* during the interaction with Nao was based on the laughter intensity scale developed by Law et al [57].

### Statistical Analyses

#### Power

The sample size was determined by a power analysis using the G*Power program created by Faul et al [58]. The following parameters were selected: an alpha error probability of .05, power of 0.80, and an effect size (f) of 0.31. The effect size used was based on an average of the effect sizes found in previous studies examining robot social behaviors in the context of human-robot interactions [14,22]. The analysis revealed a total sample size of 180 participants (45 participants per experimental condition) would be required.

#### Interaction Times

A 1-way analysis of variance (ANOVA) was used to analyze the total interaction times to determine if time spent with the Nao robot differed significantly between conditions. This analysis was undertaken as a significant difference in interaction times between groups would represent a potential confound.

#### Self-Report Measures

One-way ANOVAs were used to analyze the total participant engagement and total perceived robot empathy scores, with a posthoc (Tukey) test used to compare the conditions pairwise when an ANOVA was found to be significant. Fisher exact tests were used to analyze the results of the pair-choice engagement items. A Kruskal-Wallis test was performed to analyze total perceived robot attention because of the data being found to violate normality.

#### Video Analyses

One-way ANOVAs were used to analyze the percentage time participants spent looking at the robot (participant eye gaze) as well as the percentage time participants spent smiling during the interaction with the robot, with a posthoc (Tukey) test used to compare the conditions individually when an ANOVA was found to be significant. Fisher exact tests were used to determine if any significant differences existed between conditions in regard to whether or not participants laughed during the interaction with the robot, as well as whether or not participants leaned toward the robot during the interaction.

All analyses were performed using the SPSS, version 22.

## Results

### Manipulation Check

A manipulation check was performed using a convenience sample (n=10). Participants undertaking the manipulation check were asked to view 4 separate video recordings of the robot (1 video of each condition) and completed a brief measure after each video. The measure used asked participants to indicate whether the robot in the video demonstrated a forward lean, used self-disclosure statements, used voice pitch changes, or *none of the above*. Before viewing the recordings, participants were verbally given the following definition to identify robot self-disclosure: “self-disclosure is the act of revealing personal information about oneself to another [12], please indicate if you feel the robot has revealed personal information about itself in any of the following recordings.”

Furthermore, 100% (10/10) of participants were able to accurately identify self-disclosure and forward lean behaviors in the robot. Of 10 participants, 1 (10%) confused the voice pitch and neutral conditions, but the remaining 9 participants (90%) were able to accurately identify voice pitch changes and neutral behaviors in the robot.

### Participants

In total, 181 participants took part in this study. Participants were predominantly female (112/181, 61.9%) and ranged in age from 17 to 80 years (mean 25.8, SD 10.21). Most participants were students (n=139), followed by part-time employees (n=20), full-time employees (n=19), and those who were retired or currently unemployed (n=4). Participants mainly identified as being of New Zealand European ethnicity (n=57), followed by Chinese (n=37), Indian (n=29), Korean (n=5), Maori (n=4), Samoan (n=3), and Tongan (n=1). In addition, 49 participants identified as having an ethnicity other than those listed on the baseline questionnaire form. Most participants (148/181, 81.8%,) advised that they had never before interacted with any kind of robot.

### Interaction Times

Interactions ran from 145 to 284 seconds in total, with a mean total interaction time of 182 seconds. There were no significant differences found between the means of the neutral (187.98), forward lean (178.26), self-disclosure (180.72), and voice pitch (180.67) conditions in regard to total interaction time with the Nao robot (*F*_3,173_=1.14; *P*=.34).

### Participant Perceived Engagement Scores

A 1-way ANOVA of total participant engagement scores (*F*_3,177_=1.420; *P*=.24) found no significant difference between the means of the neutral (mean 26.96, SD 5.80), forward lean (mean 27.38, SD 5.87), self-disclosure (mean 28.22, SD 5.39), and voice pitch (mean 25.84, SD 5.26) conditions.

A Fisher exact test of pair-choice engagement items found that participants in the voice pitch condition were significantly more likely to rate Nao as boring (as opposed to interesting) compared with the neutral, self-disclosure, and forward lean groups (χ^2^_3_=10.3; *P*<.001; n=179). A large effect size (Cramer’s V=.29) was found for this item. No significant differences were found between the conditions in regard to participant rating of *unimaginative versus creative* (χ^2^_3_=2.4; *P*=.54; n=178), *cautious versus bold* (χ^2^_3_=5.6; *P*=.13; n=172), *innovative versus conservative* (χ^2^_3_=0.3; *P*=.97; n=180), *dull versus absorbing* (χ^2^_3_=5.5; *P*=.14; n=177), or *novel versus conservative* (χ^2^_3_=3.4; *P*=.34; n=178). Participants in the voice pitch and neutral conditions were significantly more likely to rate Nao as unstimulating (as opposed to stimulating) compared with the self-disclosure and forward lean groups (χ^2^_3_=8.8; *P*=.03; n=176). A medium effect size (Cramer’s V=.22) was found for this item.

### Perceived Robot Empathy

A 1-way ANOVA of total perceived robot empathy scores (*F*_3,175_=1.89; *P*=.13) found no significant differences between the means of the forward lean (mean 44.23, SD 6.72), self-disclosure (mean 43.83, SD 7.32), voice pitch (mean 41.33, SD 6.92), and neutral (mean 41.95, SD 6.38) conditions.

### Perceived Robot Attention

A Kruskal-Wallis test of total perceived robot attention scores (χ^2^_3_=1.1; *P=*.78; n=181) found no significant difference between the mean rank (MR) scores of the forward lean (MR=94.44), self-disclosure (MR=94.63), voice pitch (MR=90.02), and neutral (MR=84.82) conditions.

### Participant Behaviors

In total, 174 video recordings were coded for analysis. Of the 7 participant interactions that were not analyzed, 5 were excluded because of the technical difficulties with recording equipment, and 2 were excluded because of participants’ refusal to be recorded during the interaction.

### Participant Eye Gaze

There was a significant difference in the percentage time participants spent looking at the Nao robot during the interaction (*F*_3,173_=8.13; *P*<.001). Participants in the forward lean (mean 78.80, SD 8.98) condition spent significantly more time looking at the robot compared with participants in the neutral (mean 69.14, SD 10.96) and voice pitch (mean 73.30, SD 9.88) conditions. Participants in the self-disclosure (mean 76.30, SD 8.78) condition were found to have spent significantly more time looking at the Nao robot during the interaction compared with participants in the neutral condition. A medium to large effect size (η2=.13) was found for this condition. All other comparisons were nonsignificant.

### Participant Use of Forward Lean

There was a significant difference between the conditions in regard to whether or not participants leaned toward the Nao robot during the interaction (χ^2^_3_=22.1; *P*<.001; n=174). Significantly, more participants leaned toward the Nao robot in the forward lean condition, 67% (31/46), compared with the self-disclosure, 47% (20/42), voice pitch, 39% (17/43), and neutral, 18% (8/43) conditions. A large effect size (Cramer’s V=.36) was found for this condition.

### Participant Smiling

There were no significant differences in the percentage of time participants spent smiling during the interaction with the robot (*F*_3,173_=0.801; *P*=.50). The means of the neutral (mean 9.35, SD 9.28), forward lean (mean 9.20, SD 8.25), self-disclosure (mean 11.98, SD 9.73), and voice pitch (mean 10.70, SD 10.90) conditions did not significantly differ in regard to the percentage of time participants spent smiling during the interaction.

### Participant Laughing

There was a significant difference between groups in whether participants laughed or not (χ^2^_3_=12.0; *P=*.01; n=174). Significantly, more participants laughed in the self-disclosure condition, 47% (20/42), compared with the forward lean, 21% (10/46), voice pitch, 20% (9/43), and neutral, 18% (8/43) conditions. A medium effect size (Cramer’s V=.26) was found for this condition. 

## Discussion

### Principal Findings

The forward body lean and self-disclosure robot behaviors showed significant effects on both self-reported outcomes and observed behaviors compared with the voice pitch and neutral conditions. Participants in the robot forward body lean condition spent significantly more time looking at the robot compared with participants in the voice pitch and neutral conditions. They were also more likely to lean forward toward the robot than those who interacted with a neutral robot and reported the robot was significantly more stimulating than participants in the voice pitch and neutral conditions. Participants in the self-disclosure condition also spent significantly more time looking at the robot compared with participants in the neutral condition, and significantly, more participants in the self-disclosure condition laughed during the interaction compared with participants in the forward lean, voice pitch, and neutral conditions. The voice pitch condition had no effects or even slightly negative effects compared with the other conditions with participants in the forward lean, self-disclosure, and neutral conditions finding the robot to be significantly more interesting than participants in the voice condition.

There were no significant differences found between groups in regard to self-reported engagement, perceived robot empathy, or perceived robot attention. A potential explanation for these results may be found in the use of a between-subjects study design. This design may have resulted in a lack of comparison for the majority of participants (148/181) who had never interacted with any kind of robot before this experiment. Another potential explanation is that the novelty or excitement of a *first encounter* with a robot may have created some ceiling effects in regard to the measures used. Certainly, many participants expressed excitement in regard to interacting with the Nao robot, and the total scores for participant perceived robot attention and participant perceived engagement were positively skewed regardless of condition. Finally, some of the null outcomes may be because of the use of self-report measures that rely on the memory of the interaction.

In contrast, the study did find differences between groups in behavioral measures of attention and engagement. Differences between groups in eye gaze, laughing, and forward lean suggest that participant engagement was significantly higher in the forward lean and self-disclosure groups compared with the voice pitch and neutral conditions. Behavioral measures offer some advantages over self-reports, as they are less prone to memory and social desirability bias and can be more sensitive, valid, and reliable [59].

### Comparison With Prior Work

#### Forward Lean

This study is novel in many regards. It is the first study to investigate the use of robot forward body lean in a human-robot interaction and the first to show that robot forward body lean can positively influence users’ attentional behaviors and self-reported stimulation. This finding is salient in terms of social robotics research, as it represents a simple robot nonverbal behavior that may be used to increase user engagement. Increasing engagement between health care robots and users is fundamental to ensuring positive interactions and important in establishing user attention and comprehension. Previous literature examining interactions between health care professionals and patients demonstrates the importance of a forward lean by a clinician to demonstrate *active listening* [29]. By leaning forward toward participants when it spoke, the Nao robot may have been perceived by participants as actively listening to their questions and responses. It may be therefore that the increased eye gaze and forward body lean behaviors observed in participants in the forward body lean condition represent a form of reciprocated attention, or mirroring, toward the robot. This theory is supported by the fact that participants in the forward lean condition found the interaction with the Nao robot to be significantly more stimulating and significantly more interesting when compared with the neutral condition. As the videos in this study were not analyzed in regard to *when* forward leaning occurred, just whether or not a forward leaning behavior was observed, reciprocated attention, or mirroring, could not be ascertained.

In contrast to previous research showing an association between physician forward body lean and patient perceptions of physician empathy [39], the robot’s use of forward body lean did not result in increased user perceptions of robot empathy. As discussed above, this finding may be because of the use of a between-subjects design, resulting in a lack of comparison for the majority of participants who had never interacted with a robot before. A within-subjects design in which participants interact with both the neutral robot and forward lean robot may have shown different outcomes in regard to user perceived robot empathy.

#### Self-Disclosure

Although previous research in robotics has investigated the effect of robot self-disclosure in terms of decreasing user anxiety, increasing user perceived robot likability, and increasing user perceived robot mind attribution [12,14,15], this is the first study to show that self-disclosure by a robot can increase user attentional and engagement behaviors. Although participant *self-reported* measures reflecting *perceived* engagement did not differ significantly when comparing the self-disclosure condition with the neutral condition, participants in the self-disclosure condition did spend significantly more time *looking* at the robot during the interaction compared with participants in the neutral condition. This observed increase in robot-directed eye gaze behaviors indicated that there was an increased level of participant attention and engagement with the Nao robot in the self-disclosure condition.

Once again, this increase in user engagement gives important insights into the basic social behaviors that a social or health care robot can display to increase engagement and attention during interactions. As discussed earlier, increased robot-directed eye gaze behaviors, such as seen in this experiment, have also been found in research examining the effect of self-disclosure in human social interactions [4].

Participant laughing also indicates a higher level of engagement during the self-disclosure condition compared with the neutral condition. Participants in the self-disclosure condition were found to laugh significantly more than participants in any of the other conditions. This was an incidental finding and not one we had expected to make when beginning this experiment. Participants in this condition were most likely to laugh when the Nao robot stated, “Don’t worry, I forget things too sometimes” in response to a participant stating that they had forgotten the name of their doctor. It may be that, as well as acting as self-disclosure statement, participants perceived this statement as being humorous. The use of humor in health care environments is gaining attention as a useful therapeutic tool for the facilitation of positive patient health outcomes [60]. This in turn has generated a fledgling area of social robotics research investigating the effect of robot humor on human-robot interactions [61,62]. Given the significant response by participants (in the form of laughter) to the Nao robots *forgetfulness*, it appears that robot humor is an area worth exploring.

#### Voice Pitch

Although previous research has examined the effect of robot voice gender, robot voice age, and robot voice human likeness [30-32] in regard to user outcomes, only 1 other study has investigated robot voice pitch within the context of a human-robot interaction [22]. In this study [22], researchers used a within-subjects design to compare participant interactions between a robot using a *high* voice pitch against interactions with a robot using a *low* voice pitch, finding that the robot with the high voice pitch was rated more positively by participants. The robot in the voice pitch condition of our study was programmed to use both high *and* low voice pitch changes within a single interaction. The lack of significant results in our study in regard to the voice pitch condition may suggest that voice pitch changes are beneficial only if they are in the higher range. Another potential explanation for these findings may be inadequate frequency and/or distinction in regard to the voice pitch changes used by the robot. Indeed, the manipulation check did show that 1 of 10 participants had difficulty distinguishing the voice pitch condition from the neutral condition.

### Contribution to Existing Literature

This study demonstrates the importance of robot forward lean and self-disclosure in increasing human attentional and engagement behaviors in a health care application. The ability of a robot to attract and sustain human attention is particularity important in health care environments. Patients in these settings need to pay attention to advice, reminders, and recommendations of health care robots. The implications of this research are that social robot designers should consider the implementation of robot forward lean and self-disclosure to increase user attention, particularly with regard to health care robots. These results extend the literature demonstrating the importance of forward lean and self-disclosure in interactions between health professionals and patients to robots [38-41]. Furthermore, this research supports a robot-patient interaction model that proposes the importance of verbal and nonverbal behaviors to user outcomes [16].

### Limitations

Similar to many studies in human-robot interaction, the participants were mainly students and relatively young. Younger people may be more positive and open in regard to interacting with a robot than older people, and therefore, the results may have limited generalizability to an older population. In addition, the study was conducted in a laboratory setting, and further research is needed in a more realistic setting. Another limitation is that the study used a scripted interaction. The behaviors and self-reported measures observed may have differed from what would have been observed had a natural conversation taken place between participants and the Nao robot. Finally, there is research to suggest that an individual’s personality type may influence perceptions and attitudes toward robots [63]. As we did not use any measures of human personality type in this study, it is unknown whether participants’ personality influenced outcomes. Nevertheless, randomization to groups should ensure that personality did not systematically differ between groups. A strength of the research is that participants were blinded to group allocation.

### Future Work

Future research could consider the use of a within-subjects design to provide participants with a basis of comparison, allowing for greater insight into the effects of specific robot attentional behaviors within an experimental context. Furthermore, the use of natural speech, rather than a scripted interaction, may allow participants to concentrate fully on the interaction with the robot, as opposed to splitting attention between the robot and script.

Given that the real-life implementation of a robot medical receptionist would certainly take place within a natural environment (such as a medical clinic), research exploring the use of a health care robot’s attentional behaviors within the context of such an environment (eg, an actual doctor’s clinic) is necessary. This research would not only provide potentially significant insight into the effect of robot attentional behaviors in naturalistic settings but may also allow for recruiting of a more mixed-age sample.

Finally, research investigating the effect of robot voice pitch changes in human-robot interactions may need to focus on using more distinct and/or frequent voice pitch changes to see significant effects. Future work could accentuate a health care robot’s voice pitch changes and explore the effects on user perceptions of robot acceptability.

### Conclusions

This study explored the effects of robot self-disclosure, robot forward lean, and robot voice pitch changes on user perceptions of engagement, robot attention, and robot empathy, as well as user attentional behaviors. Robot self-disclosure and forward body lean resulted in significantly better self-reported outcomes and observed behaviors compared with the neutral condition. Robot voice pitch changes did not have positive effects, but more research is needed to further investigate this. Exploring the effect of human social behaviors, such as attentional behaviors, within the context of human-robot interactions in health care, represents a salient opportunity to inform future robotic design and implementation.

## Appendix

Multimedia Appendix 1 Participant scenario information sheet.

Multimedia Appendix 2 Postinteraction measures.

Multimedia Appendix 3 CONSORT‐EHEALTH checklist (V 1.6.1).

## Abbreviations

ANOVA: analysis of variance
MR: mean rank
