# Correction: The Effect of Robot Attentional Behaviors on User Perceptions and Behaviors in a Simulated Health Care Interaction: Randomized Controlled Trial

**DOI:** 10.2196/18362

**Published:** 2020-03-03

**Authors:** Deborah L Johanson, Ho Seok Ahn, Bruce A MacDonald, Byeong Kyu Ahn, JongYoon Lim, Euijun Hwang, Craig J Sutherland, Elizabeth Broadbent

**Affiliations:** 1 Department of Psychological Medicine Faculty of Medical and Health Sciences The University of Auckland Auckland New Zealand; 2 Centre for Automation and Robotic Engineering Science The University of Auckland Auckland New Zealand; 3 Department of Electrical, Computer, and Software Engineering Faculty of Engineering The University of Auckland Auckland New Zealand

The authors of “The Effect of Robot Attentional Behaviors on User Perceptions and Behaviors in a Simulated Health Care Interaction: Randomized Controlled Trial” (J Med Internet Res 2019;21(10):e13667) noticed a typo in the abstract of their article. The Abstract should have detailed 4 groups rather than 3. In the Abstract, the Methods section has been revised from:

A parallel randomized controlled trial with a 1:1:1 allocation ration was conducted.

to:

A parallel randomized controlled trial with a 1:1:1:1 allocation ratio was conducted.

The correction will appear in the online version of the paper on the JMIR website on March 3, 2020, together with the publication of this correction notice. Because this was made after submission to PubMed, PubMed Central, and other full-text repositories, the corrected article has also been resubmitted to those repositories.

