# Supplementary material for: The Effect of Robot Attentional Behaviors on User Perceptions and Behaviors in a Simulated Health Care Interaction: Randomized Controlled Trial
Source: J Med Internet Res. 2019 Oct 4;21(10):e13667. doi: 10.2196/13667 (PMC6914232; doi:10.2196/13667)
Supplement: Multimedia Appendix 1 [file jmir_v21i10e13667_app1.pdf]

Participant Scenario Information Sheet

1) Please begin the interaction by saying "hello" to Nao.

Nao will greet you and ask how she may help you.

2) Say **"I'm here to pick up my prescription"**

When Nao asks for your name please say **"Sam Smith"**

When Nao asks for your address please say **"12 Green Street"**

Nao will inform you in regards to your prescription. She will ask if there is anything else she can do to help you.

3) Say **"I need to check in for my Doctors appointment"**

When Nao asks what time your appointment is, please say **"4pm"**

When Nao asks what Doctor you are seeing, please tell her **"I can't remember"**

Nao will check you in for your Doctor's appointment. She will ask if there is anything else she can help you with.

4) Say **"How long will I have to wait?"**

Nao will tell you the wait time to see the Doctor and ask if there is anything else she can help you with.

5) Say **"Where is the bathroom"**

Nao will give you directions to the bathroom.

6) Say: **"Okay".**

Nao will wish you a pleasant day.
