# Supplementary material for: The Effect of Robot Attentional Behaviors on User Perceptions and Behaviors in a Simulated Health Care Interaction: Randomized Controlled Trial
Source: J Med Internet Res. 2019 Oct 4;21(10):e13667. doi: 10.2196/13667 (PMC6914232; doi:10.2196/13667)
Supplement: Multimedia Appendix 2 [file jmir_v21i10e13667_app2.pdf]

Participant Experience Tool

Please think about the interaction you just had with Nao and answer the following questions:

How engaging did **YOU** find the interaction with Nao?
How exciting was the interaction with Nao?
How completely were your senses engaged during the interaction with Nao?

Please consider your interaction with Nao and indicate how true the following statements are for you:

"I was so involved in the interaction, I lost track of time".
"The experience caused real feelings or emotions for me".
"I had fun interacting with Nao".
"The interaction with Nao was like interacting with a real person"
"Nao had appropriate eye contact"

Please circle ONE of EACH of the following pairs:

"**I** found the interaction with Nao to be...":

Interesting	Boring
Unimaginative	Creative
Bold	Cautious
Innovative	Conservative
Dull	Absorbing
Unstimulating	Stimulating
Novel	Conventional

Perceived Robot Empathy Questionnaire

Please consider your interaction with Nao and indicate how true the following statements are for you:

Nao made me feel at ease
Nao showed care and compassion
Nao had a positive approach
Nao explained things clearly
Nao really listened to me
Nao helped me during our interaction
Nao's comments were useful to me
Naos comments were helpful to me
If I were worried, Nao would make me feel better
If I were nervous, Nao would make me feel more calm
If I were upset, Nao would make me feel better
I feel Nao paid attention to me

Perceived Robot Attention

Please think about the interaction you just had with Nao and answer the following questions:

How engaged do you think **NAO** was during the interaction?
How completely do you feel Nao listened to you during the interaction?

Please consider your interaction with Nao and indicate how true the following statements are for you:

"Nao was completely engaged during the interaction".
"Nao listened to me and understood what I was saying".
"I think Nao had fun during our interaction".
